# Quercetin-Induced Lifespan Extension in *Podospora anserina* Requires Methylation of the Flavonoid by the *O*-Methyltransferase PaMTH1

**DOI:** 10.3389/fgene.2018.00160

**Published:** 2018-05-04

**Authors:** Verena Warnsmann, Saskia Hainbuch, Heinz D. Osiewacz

**Affiliations:** Molecular Developmental Biology, Institute of Molecular Biosciences and Cluster of Excellence Frankfurt Macromolecular Complexes, Department of Biosciences, J. W. Goethe University, Frankfurt, Germany

**Keywords:** *Podospora anserina*, lifespan, quercetin, methyltransferase, isorhamnetin

## Abstract

Quercetin is a flavonoid that is ubiquitously found in vegetables and fruits. Like other flavonoids, it is active in balancing cellular reactive oxygen species (ROS) levels and has a cyto-protective function. Previously, a link between ROS balancing, aging, and the activity of *O*-methyltransferases was reported in different organisms including the aging model *Podospora anserina.* Here we describe a role of the *S*-adenosylmethionine-dependent *O*-methyltransferase PaMTH1 in quercetin-induced lifespan extension. We found that effects of quercetin treatment depend on the methylation state of the flavonoid. Specifically, we observed that quercetin treatment increases the lifespan of the wild type but not of the *PaMth1* deletion mutant. The lifespan increasing effect is not associated with effects of quercetin on mitochondrial respiration or ROS levels but linked to the induction of the *PaMth1* gene. Overall, our data demonstrate a novel role of *O*-methyltransferase in quercetin-induced longevity and identify the underlying pathway as part of a network of longevity assurance pathways with the perspective to intervene into mechanisms of biological aging.

## Introduction

One of the oldest dreams of the human species is the dream of a long and healthy life. In search of the “fountain of youth,” diverse strategies for interventions into aging processes have been suggested. Among others, these are regular exercise and different types of diets. For instance, caloric restriction is well known in different species to lead to longevity and better health in old age (Fontana et al., 2010). Another one is a mediterranean type of diet rich in fruits, vegetables, and olive oil (Chrysohoou et al., 2004; Tyrovolas and Panagiotakos, 2010; Bach-Faig et al., 2011).

A main goal of current aging research is to identify specific active components in complex diets and to elucidate the mechanisms by which they lead to healthy aging. As part of this goal, components of nutrients, which are considered to promote healthy life, are investigated. Polyphenols are such substances, which represent one of the largest and ubiquitous groups of secondary metabolites found in fruits and vegetables. With more than 6000 known members, flavonoids are the largest subgroup of polyphenols (Manach et al., 2005). It is known that flavonoids are excellent scavengers of reactive oxygen species (ROS) (Bors et al., 1994). Apart from this function as antioxidants, flavonoids can also act as pro-oxidants at least under some circumstances as in the presence of a high concentrations of free transition metal ions (Canada et al., 1990; Decker, 1997; Croft, 1998; Knab and Osiewacz, 2010) or high doses of flavonoids (Yen et al., 2003; Wätjen et al., 2005). In general, flavonoids affect the abundance of ROS, molecules that, in low abundance, are essential for cell signaling to control healthy development. At high concentrations, due to their reactivity, ROS lead to damage of all kinds of cellular components and to degenerative processes like disease and aging. Such negative effects of ROS have been conceptualized in the “free radical theory of aging” (FRAT) and the “mitochondrial free radical theory of aging” (MFRAT) (Harman, 1956, 1972). These theories paved the road for aging research for decades. For instance, an increased ROS scavenging was found to increase organismic lifespan of *Drosophila melanogaster* (Agarwal and Sohal, 1994; Sohal et al., 1995), *Caenorhabditis elegans* (Larsen, 1993), *Podospora anserina* (Zintel et al., 2011), and to increase chronological lifespan of *Saccharomyces cerevisiae* (Harris et al., 2003).

A link between the anti-oxidative capacity, aging, and lifespan control was also demonstrated in studies with quercetin, the major representative of flavonoids. In studies with different model systems, a ROS scavenging potential of quercetin was observed (Kampkötter et al., 2007, 2008; Nabavi et al., 2012). For instance, the anti-oxidative capacity of quercetin can be linked to lifespan extension in *C. elegans*, *S. cerevisiae*, and *D. melanogaster* (Belinha et al., 2007; Kampkötter et al., 2008; Saul et al., 2008; Pietsch et al., 2009). Furthermore, Niklas et al. (2012) first reported an increased longevity in human cell cultures by quercetin treatment. Beside the anti-oxidative capacity, studies with cell cultures and rats identified a pro-oxidative effect of quercetin (Laughton et al., 1989; Metodiewa et al., 1999; Robaszkiewicz et al., 2007). *In vitro* studies demonstrated that the anti-oxidative as well as the pro-oxidative function of quercetin is attributed to the characteristic catechol group containing two vicinal hydroxyl residues (3′- or 4′-hydroxyl residues) (Heijnen et al., 2002). *O*-Methylation (from now on termed methylation) of these residues can prevent both functions (Cao et al., 1997; Lemanska et al., 2004; Duenas et al., 2010). In humans and rodents, it was shown that this kind of methylation is catalyzed by catechol-*O*-methyltransferases, a specific group of *O*-methyltransferases (Zhu et al., 1994, 2010; Chen et al., 2011).

Also in *P. anserina*, an aging model with a short lifespan that is accessible to genetic and molecular manipulation (Scheckhuber and Osiewacz, 2008; Wiemer et al., 2016) a protein, which accumulates during aging in total protein extracts, has been identified. This protein turned out to be an *O*-methyltransferase (PaMTH1) with significant homology to the catechol-*O*-methyltransferases of humans, rodents, and other species (Averbeck et al., 2000). *In vitro* studies revealed that flavonoids are potential substrates of PaMTH1. An activity assay with PaMTH1 recognized myricetin, quercetin, and other flavonoids with vicinal hydroxyl residues as potential substrates (Kunstmann and Osiewacz, 2008). NMR spectroscopy confirmed that PaMTH1 has the ability to methylate myricetin and that PaMTH1 is active as a dimer (Chatterjee et al., 2015). Furthermore, *in vivo* studies revealed an age-related PaMTH1 accumulation not only in total cell extracts but also in mitochondria (Averbeck et al., 2000; Groebe et al., 2007; Kunstmann and Osiewacz, 2008).

A connection of oxidative effects and methyltransferase function was demonstrated in *P. anserina.* Overexpression of *PaMth1* was shown to lead to a reduced protein carbonylation suggesting that the methylation of PaMTH1 substrates prevents the incomplete oxidation and thereby the formation of ROS (Kunstmann and Osiewacz, 2008). This protective role of PaMTH1 was confirmed in investigations with a *PaMth1* deletion mutant, which showed a decreased oxidative stress resistance (Kunstmann and Osiewacz, 2009). Such a link between *O*-methyltransferase activity and aging was also obtained in other organisms. For instance, in rats a link between an *O*-methyltransferase and age-related repair of damaged proteins was demonstrated (Shirasawa et al., 1995). In humans an increase of the abundance and activity of an *O*-methyltransferase during aging was reported (Tunbridge et al., 2007). It appears that methylation of substrates by *O*-methyltransferase is part of evolutionary conserved pathways which affect organismal aging. However, the detailed underlying mechanisms are still poorly elucidated.

In a series of recent studies, we started to investigate the impact of natural compounds on *P. anserina* aging. Among others, we studied the polyphenols gossypol (Warnsmann et al., 2017), curcumin (Warnsmann and Osiewacz, 2016), and here we investigated quercetin. We report novel details about the impact of quercetin on lifespan in *P. anserina*. We demonstrate that quercetin supplementation leads to an increased abundance of the PaMTH1 *O*-methyltransferase and that quercetin-induced lifespan extension requires the methylation of this flavonoid by PaMTH1. Furthermore, we reveal effects on respiration and ROS levels attributed to unmethylated quercetin which are not necessary for lifespan control.

## Materials and Methods

### *P. anserina* Strains and Cultivation

In this study, the *P. anserina* wild-type (WT) strain “s” (Rizet, 1953) and the previously generated strains *ΔPaMth1* (Kunstmann and Osiewacz, 2009) and *PaMth1_OEx* (Kunstmann and Osiewacz, 2008) were used. All transgenic strains are in the genetic background of the WT strain “s.” Strains were grown on standard cornmeal agar (BMM) at 27°C under constant light (Osiewacz et al., 2013). For spore germination, standard cornmeal agar (BMM) with 60 mM ammonium acetate was used and incubated at 27°C in the dark for 2 days (d). All strains of this study were derived from monokaryotic ascospores (Osiewacz et al., 2013).

### Quercetin and Isorhamnetin Supplementation

After spore germination, all subsequent cultivating steps were performed on M2 medium (Osiewacz et al., 2013) with a supplementation by 300 μM of quercetin (Sigma, Q4951) or 300 μM isorhamnetin (Roth, 6528.1). Additionally, as a control, M2 medium was supplemented with 0.15% dimethylsulfoxide (DMSO; Roth, 4720.1).

### Growth Rate and Lifespan Determination

Determination of the lifespan and the growth rate of *P. anserina* cultures derived from monokaryotic ascospores were performed on M2 medium with or without supplements at 27°C and constant light as previously described (Osiewacz et al., 2013). The lifespan of *P. anserina* is defined as the time period in days (d) of linear hyphal growth while the growth rate is defined as the measured growth (cm) per time period (d).

### Isolation of Mitochondria

*P. anserina* strains were grown on cellophane foil covered solid M2 medium containing different supplements for 2 d at 27°C and constant light. Mycelial pieces were transferred to CM-liquid medium containing different supplements and grown at 27°C and constant light for additional 2 d. Mitochondria of *P. anserina* cultures was isolated as previously described by differential centrifugation for measurement of mitochondrial oxygen consumption and by discontinuous sucrose gradient (20–36–50%) ultracentrifugation for blue-native polyacrylamide gels (BN-PAGE) analysis (Osiewacz et al., 2013).

### Mitochondrial Oxygen Consumption

Determination of mitochondrial oxygen consumption was performed at 27°C by high-resolution respirometry (Oxygraph-2k series C and G, OROBOROS Instruments, Innsbruck, Austria). 200 μg freshly prepared mitochondria was injected into 2 ml air saturated oxygen buffer (0.3 M sucrose, 10 mM KH_2_PO_4_, 5 mM MgCl_2_, 1 mM EDTA, 10 mM KCl, and 0.1% BSA; pH 7.2). To promote the ADP-limited complex I-dependent state 4 respiration (state 4) 10 mM pyruvate (Sigma-Aldrich, P2256) and 2 mM malate (Sigma-Aldrich, M1000) were added. Subsequently, 1.5 mM ADP (Sigma-Aldrich, A5285) was added to determine complex I-dependent state 3 respiration (state 3). Data were analyzed using the manufacturer’s software DatLab 6.

### Blue-Native Polyacrylamide Gels (BN-PAGE)

Blue-native polyacrylamide gels was performed as previously described (Wittig et al., 2006). For sample preparation, 100 μg of mitochondrial protein extracts were solubilized using a digitonin (Sigma-Aldrich, D141) protein ratio of 3:1 (w/w). Linear gradient gels (4–13%) overlaid with 3.5% stacking gels were used for separation of the solubilized mitochondrial protein extracts. Respiratory chain components were visualized by Coomassie blue staining and assigned as described previously (Krause et al., 2004).

### Determination of Superoxide Release

Qualitative determination of superoxide release from mycelia was performed by monitoring the reduction of nitroblue tetrazolium (NBT, Sigma-Aldrich, N6876) using a modified protocol of Munkres (1990). Briefly, *P. anserina* strains were cultivated for 4 d on M2 agar medium with the different supplements in the dark at 27°C. The plates were floated with 5 ml staining solution for superoxide (contains 5 mM MOPS pH 7.6, 2.5 mM NBT) and incubated for 30 min in the dark at 27°C. The solution was decanted, the plates were incubated for additional 3 h in the dark, and at 27°C to obtain the desired staining intensity.

### Hydrogen Peroxide Release Measurements

Qualitative determination of hydrogen peroxide release from mycelia was performed by monitoring the oxidation of diaminobenzidine (DAB, Sigma-Aldrich, D-8001) according to a modified protocol of Munkres (1990). *P. anserina* strains were cultivated for 4 d on M2 agar medium with the different supplements in the dark and 27°C. The plates were floated with 5 ml staining solution for hydrogen peroxide (contains 100 mM Tris/HCl pH 6.9, 2.5 mM DAB; dissolved at 60°C for 10 min) and incubated for 30 min in the dark and 27°C. The solution was decanted, the plates were incubated for additional 3 h in the dark, and 27°C to obtain the desired staining intensity. Quantitative measurement of hydrogen peroxide was performed as previously described (Kowald et al., 2012).

### Isolation of Total Protein Extract

Isolation of total protein extract was performed as previously described (Osiewacz et al., 2013). Like for mitochondria isolation, appropriate supplements were added to M2 agar and to CM-liquid media.

### “In-Gel” SOD Activity Assay

“In-gel” SOD activity assays were performed as described (Osiewacz et al., 2013).

### “In-Gel” Peroxidase and Catalase Activity Assay

“In-gel” peroxidase and catalase activity assays were performed as described in a previously published protocol (Wayne and Diaz, 1986).

### Western Blot Analysis

Separation of 100 μg total protein extract by SDS–PAGE and following transfer of proteins to PVDF membranes (Immobilon-FL, Millipore) was performed according to standard protocols (Knuppertz et al., 2014). Blocking and antibody incubation of blotted PVDF membranes were performed in accordance to the Odyssey “Western Blot Analysis” handbook (LI-COR). Primary antibodies were raised against a PaSOD2-specific synthetic peptide ([Ac]-CERFLGTSEATKL[OH]; New England Peptide; 1:2000 dilution) corresponding to AA 225–236 and against a PaMTH1-specific synthetic peptide (PFNEETADRVSAYC-KLH; Sigma; 1:2000 dilution with total protein extract and 1:1000 dilution with mitochondrial protein extract). Additionally, following commercial available antibodies were used: a polyclonal rat MnSOD antibody (Biomal Stressgen, SOD-111, dilution: 1:2000) was used to detect *P. anserina* mitochondrial superoxide dismutase (SOD) (PaSOD3) and a polyclonal Cu/ZnSOD antibody (Biomol Stressgen, SOD-100; 1:2000 dilution) was used for detection of the cytoplasmic SOD (PaSOD1). Subsequently, secondary antibodies with the infrared dye IR Dye 800 (dilution: 1:15,000, Li-COR Biosciences) or IR Dye 680 LT (dilution: 1:20,000, Li-COR Biosciences) were used. For detection and densitometric quantification, the “Odyssey Infrared Imaging System” (Li-COR Biosciences) was used.

### Statistical Analysis

For statistical analysis of lifespans, the IBM SPSS statistics 19 software package was used and Kaplan–Meier survival estimates generated. Significances were determined with pairwise comparison with three independent statistical tests [Breslow (generalized Wilcoxon), Log Rank (Mantel–Cox), and the Tarone–Ware]. In the figure legends only the *P*-value calculated with the Breslow test is given, all other values are in the supplements (Supplementary Table S1). For all other statistical analysis, the two-tailed Student’s *t*-test was used. The minimum level of statistical significance was set at *P* ≤ 0.05. ^∗^*P* ≤ 0.05, ^∗∗^*P* ≤ 0.01, ^∗∗∗^*P* ≤ 0.001.

## Results

### Quercetin Leads to an Increased Lifespan of *P. anserina*

**FIGURE 1:**
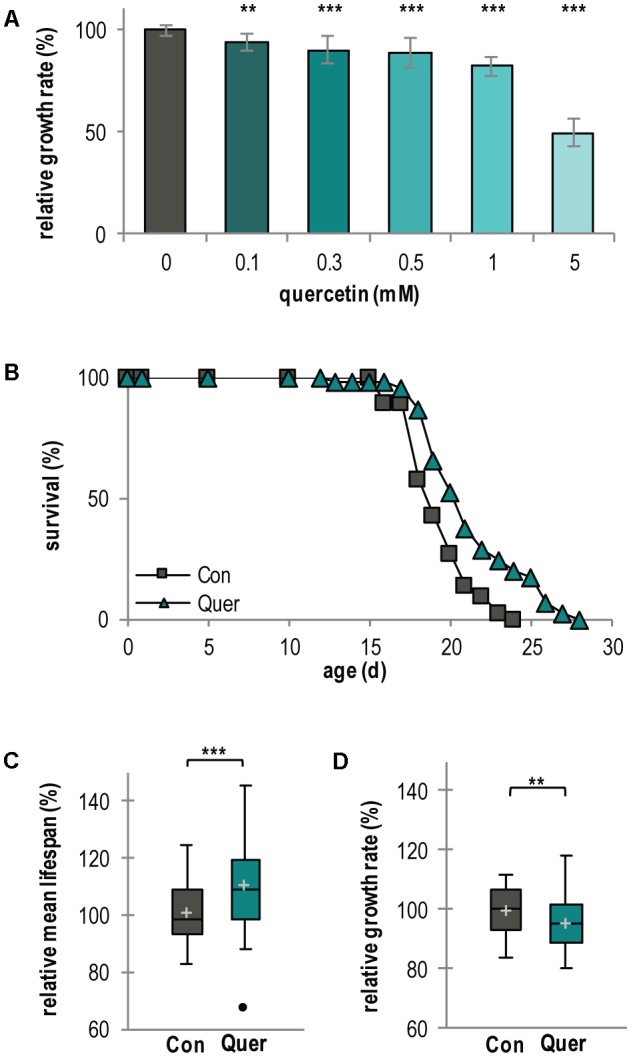
Lifespan of *P. anserina* WT is increased by quercetin treatment. **(A)** Growth rate of *P. anserina* WT strains on M2 medium containing increasing concentrations of quercetin as indicated (*n* = 10 for each concentration). **(B)** Survival curve of *P. anserina* WT cultures grown on standard M2 medium containing the solvent DMSO as control (Con; total *n* = 45) or quercetin (Quer; total *n* = 46; *P* = 0.00085). **(C)** The mean lifespan and **(D)** the mean growth rates of *P. anserina* WT cultures treated with quercetin are shown as relative values to the DMSO control. Error bars correspond to the standard deviation. The *P*-values of the survival curves were determined by SPSS with three different tests and for mean lifespan and growth rate by two-tailed Student’s *t*-test. ^∗∗^*P* < 0.01, ^∗∗∗^*P* < 0.001.

During the course of investigations to elucidate the mechanistic effects of natural compounds on organismal aging and lifespan control, we focused our interest on the polyphenol quercetin. We used the fungal aging model *P. anserina* (Scheckhuber and Osiewacz, 2008) which is a good model system for these kind of studies (Warnsmann and Osiewacz, 2016; Warnsmann et al., 2017). First, we analyzed the effect of increasing quercetin concentrations on the growth rate of the fungus and found a concentration-dependent growth rate decrease. A concentration of 5 mM was found to reduce the growth rate by 50% whereas 300 μM lead to a 10% decrease (**Figure 1A**). These results demonstrate that exogenous quercetin is taken up by *P. anserina* filamentous cells (hyphae) and that the effects of the polyphenol are concentration dependent.

Next, we investigated the effect of quercetin on lifespan in some more detail. We choose 300 μM quercetin and as control medium containing 0.15% of the solvent DMSO, a concentration that was also used in all further investigations. Under these conditions, quercetin moderately affects growth while DMSO does neither influences lifespan nor the growth rate (Supplementary Figures S1A–C). Treatment of the *P. anserina* WT resulted in a mean lifespan increase of 10.2% (21 d vs. 19 d) and a maximal lifespan extension of 16.6% (28 d vs. 24 d) (**Figures 1B,C** and Supplementary Figure S2A). As expected, the growth rate is only slightly reduced (**Figure 1D** and Supplementary Figure S2B).

### Mitochondrial Respiration and Respiratory Complexes Are Increased by Quercetin

**FIGURE 2:**
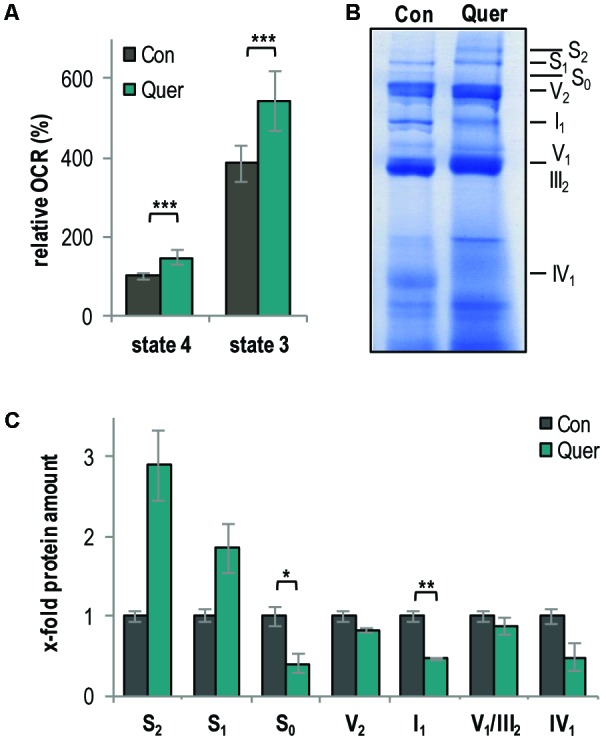
Quercetin increases respiration and the abundance of respiratory supercomplexes in the *P. anserina* WT. **(A)** Complex I-dependent oxygen consumption rate (OCR) of mitochondria from WT cultures treated with quercetin (Quer, *n* = 4 biological replicates, each with four to seven technical replicates) compared to mitochondria from cultures treated with DMSO (Con; *n* = 4 biological replicates, each with three to five technical replicates). State 4 OCR of DMSO-treated mitochondria is set to 100%. **(B)** Representative BN-PAGE analysis of isolated mitochondria from WT cultures treated with quercetin (Quer) compared to DMSO (Con)-treated cultures (*n* = 3 biological replicates for each treatment). The CI_1_CIII_2_CIV_0-2_ (S_0-2_) supercomplexes, dimeric complexes III and V (III_2_ and V_2_) as well as monomeric complexes I_1_, IV_1_, and V_1_ were visualized by Coomassie staining. **(C)** Densitometric quantification of relative protein levels of ETC complexes and supercomplexes in mitochondrial extracts from quercetin-treated cultures compared to DMSO-treated cultures. Optical densities of the different complexes were normalized to the total Coomassie staining of the corresponding lane. The mean abundance in the DMSO control was set to 1. Error bars correspond to standard deviation. The *P*-values were determined by two-tailed Student’s *t*-test. ^∗^*P* < 0.05, ^∗∗^*P* < 0.01, ^∗∗∗^*P* < 0.001.

Since a link between lifespan, mitochondrial function, and quality control in *P. anserina* and other fungi is well documented (Francis et al., 2007; Scheckhuber et al., 2011; Adam et al., 2012; Kurashima et al., 2013; Fischer et al., 2015) and since we found that other polyphenols (i.e., curcumin and gossypol) affect mitochondrial respiration in *P. anserina* (Warnsmann and Osiewacz, 2016; Warnsmann et al., 2017), we next analyzed the effect of quercetin on mitochondrial function. First, we measured the oxygen consumption rate (OCR) of isolated mitochondria by high-resolution respirometry. The complex I-dependent OCR was determined using mitochondria isolated from WT cultures treated with quercetin and DMSO, respectively. Compared to the DMSO control, quercetin significantly increased both state 4 (ADP-limiting conditions) and state 3 OCR (addition of ADP) (**Figure 2A**).

An increased respiration often results from the increased abundance of mitochondrial respiratory supercomplexes (mtRSCs) (Schägger and Pfeiffer, 2000; Bianchi et al., 2004; Genova and Lenaz, 2013). Therefore, we next analyzed the composition of the mitochondrial respiratory chain by BN-PAGE analysis and found changes. In particular, quercetin supplementation increased the abundance of the mtRSCs S_1_ and S_2_ and reduced the abundance of the mtRSC S_0_ and of monomeric complexes I and IV (**Figures 2B,C**).

### Superoxide Anion Release Is Increased by Quercetin

**FIGURE 3:**
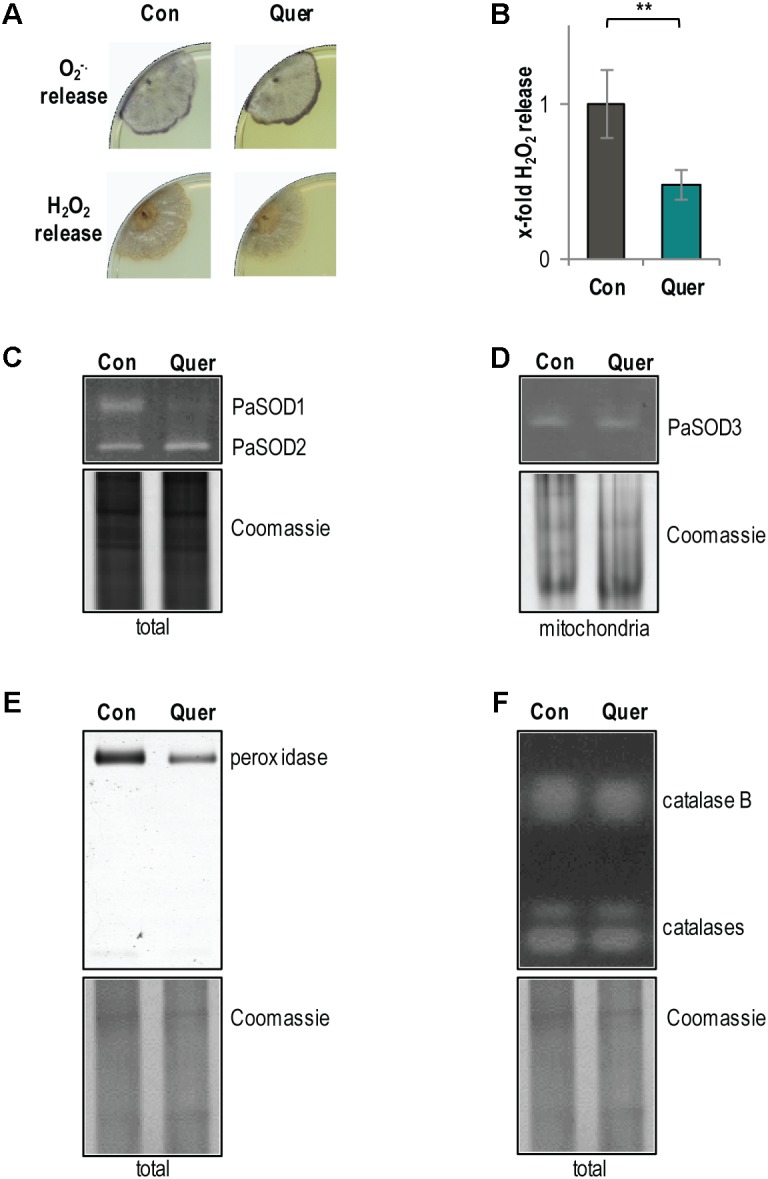
ROS metabolism of the *P. anserina* WT is affected by quercetin. **(A)** Qualitative determination of superoxide anion release by a histochemical NBT staining and hydrogen peroxide release by a histochemical DAB staining in *P. anserina* WT cultures (*n* = 4) treated with quercetin (Quer) or DMSO (Con). **(B)** Quantitative measurement of H_2_O_2_ release from *P. anserina* WT cultures treated with quercetin (Quer; *n* = 5) compared with DMSO (Con; *n* = 5) treatment. The mean release of the DMSO-treated WT was set to 1. Error bars correspond to the standard deviation and *P*-value was determined by two-tailed Student’s *t*-test. ^∗∗^*P* < 0.01. **(C)** Representative “in-gel” superoxide dismutase (SOD) (PaSOD1 and PaSOD2) activity staining from total protein extract of *P. anserina* WT cultures treated with quercetin (Quer; *n* = 4) or DMSO (Con; *n* = 4). **(D)** Representative “in-gel” SOD (PaSOD3) activity staining from mitochondrial protein extract of *P. anserina* WT cultures treated with quercetin (Quer; *n* = 3) or DMSO (Con; *n* = 3). **(E)** Representative “in-gel” peroxidase and **(F)** catalase activity staining from total protein extracts of *P. anserina* WT cultures treated with quercetin (Quer; *n* = 4) or DMSO (Con; *n* = 4).

The catechol group of quercetin contains two vicinal hydroxyl residues, which can lead to ROS production (Knab and Osiewacz, 2010). In addition, an altered respiration should also affect ROS production. Therefore, we next analyzed the production and detoxification of ROS, respectively. First, we histochemically measured the release of superoxide anions and hydrogen peroxide (Munkres, 1990) from WT cultures treated with DMSO and quercetin. Changes in the release of the superoxide anion and of hydrogen peroxide by *P. anserina* cultures are indirect measures of the ROS level in different cellular compartments including the cytoplasm and mitochondria. We observed an increase in superoxide anion (increased precipitate specifically at the growth front) and a decrease in hydrogen peroxide release (slight decrease of precipitate in the whole colony) (**Figure 3A**). The hydrogen peroxide release was subsequently confirmed by a quantitative photometric analysis. Consistently, we observed a significant decrease of hydrogen peroxide in cultures treated with quercetin compared to DMSO (**Figure 3B**).

The observed increase in superoxide anion release may be caused by an increased production or a reduced detoxification of superoxide anions. Accordingly, we analyzed the activity of superoxide anion and hydrogen peroxide converting enzymes, respectively. Activity of the three known SOD isoforms of *P. anserina* was investigated by “in-gel” staining. Compared to the DMSO control, we found a strong decrease in cytosolic PaSOD1 activity in quercetin-treated WT cultures (**Figure 3C**). The activity of the secreted PaSOD2 (Zintel et al., 2010) (**Figure 3C**) and of the mitochondrial PaSOD3 (Zintel et al., 2010) (**Figure 3D**) did not change. Consistent with previous findings showing that PaSOD1, PaSOD2, and PaSOD3 are regulated post-translationally (Borghouts et al., 2002; Wiemer and Osiewacz, 2014), we found no significant differences in the protein amount of all three isoforms upon quercetin treatment (Supplementary Figure S3). In addition, compared to the DMSO control, the activity of the hydrogen peroxide scavenging enzyme peroxidase was reduced after quercetin treatment (**Figure 3E**), whereas the activity of catalases, another class of hydrogen peroxide scavenging enzymes, was not affected (**Figure 3F**). Overall, these data indicate that the ROS detoxification system is impaired by quercetin treatment what certainly contributes to an increased superoxide anion abundance in the *P. anserina* WT.

### The PaMTH1 Level in *P. anserina* Is Increased by Quercetin

**FIGURE 4:**
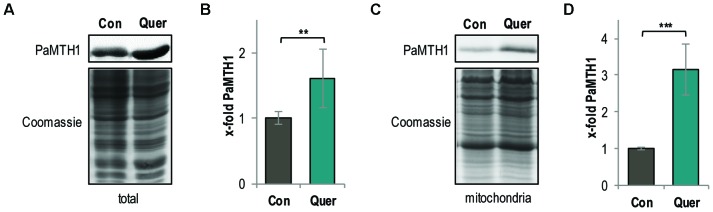
Total methyltransferase level is increased by quercetin treatment in the *P. anserina* WT. **(A)** Representative western blot analysis of total protein extract of *P. anserina* WT cultures treated with DMSO (Con; *n* = 10 biological replicates) and quercetin (Quer; *n* = 10 biological replicates), respectively. **(B)** Quantification of PaMTH1 protein level normalized to the Coomassie-stained gel. Protein level in the DMSO control was set to 1. **(C)** Representative western blot analysis of mitochondrial protein extract of *P. anserina* WT cultures treated with DMSO (Con; *n* = 6 biological replicates) or quercetin (Quer; *n* = 6 biological replicates). **(D)** Quantification of PaMTH1 protein abundance normalized to the Coomassie-stained gel. Protein level in the DMSO control was set to 1. Error bars correspond to the standard deviation and *P*-values were determined by two-tailed Student’s *t*-test. ^∗∗^*P* < 0.01, ^∗∗∗^*P* < 0.001.

Since it is known that methylation of vicinal hydroxyl groups from polyphenols affects the generation of ROS (Cao et al., 1997; Lemanska et al., 2004; Duenas et al., 2010; Knab and Osiewacz, 2010) and since previous “*in vitro*” studies revealed that quercetin is a potential substrate of the PaMTH1 *O*-methyltransferase (Kunstmann and Osiewacz, 2008), we next analyzed whether quercetin impacts the abundance of PaMTH1. Indeed, compared to the DMSO control, we found a 1.6-fold increase of the PaMTH1 abundance in total protein extract of the WT upon quercetin treatment (**Figures 4A,B**). Moreover, previous studies demonstrated an increased abundance and a translocation of PaMTH1 from the cytosol to mitochondria during aging (Averbeck et al., 2000; Groebe et al., 2007; Kunstmann and Osiewacz, 2008). Therefore, we analyzed the abundance of PaMTH1 in mitochondrial protein extracts from quercetin-treated WT cultures. Compared to the DMSO control, we found a 3.2-fold increase in mitochondria of treated cultures (**Figures 4C,D**). These results indicate that quercetin stimulates the PaMTH1 and its translocation into mitochondria.

### PaMTH1 Is Required for Quercetin-Induced Longevity

**FIGURE 5:**
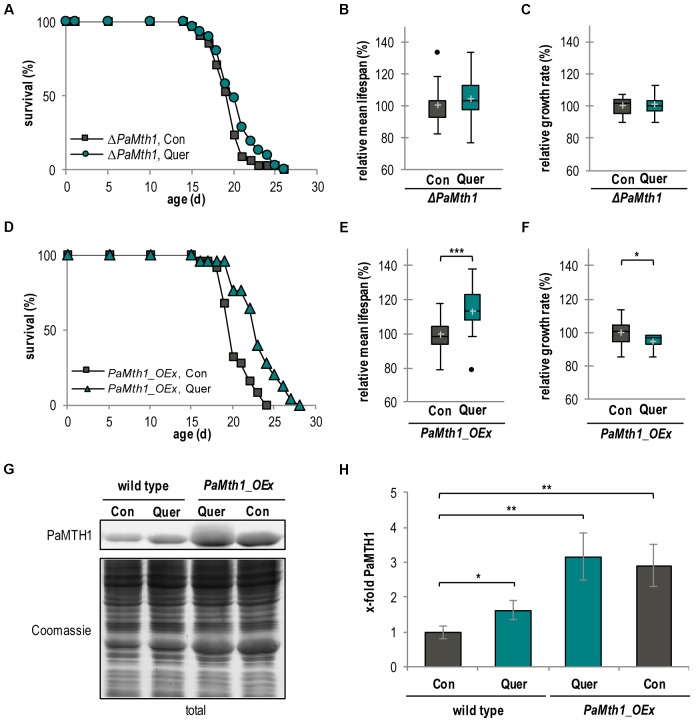
Lifespan extension by quercetin is dependent of *O*-methyltransferase activity. **(A)** Lifespan of *ΔPaMth1* cultures grown on standard M2 medium containing DMSO (Con; *n* = 34) or quercetin (Quer; *n* = 31; *P* = 0.13878). **(B)** Relative mean lifespan of cultures from **(A)**. Data shown as relative values compared to the DMSO control, which was set to 100%, ±standard derivation. **(C)** Relative mean growth rate of cultures from **(A)**. Data shown as relative values compared to the DMSO control, which was set to 100%, ±standard derivation. **(D)** Lifespan of *P. anserina Mth1_OEx* cultures grown on standard M2 medium containing DMSO (Con; *n* = 25) or quercetin (Quer; *n* = 25; *P* = 0.00012). **(E)** Relative mean lifespan of cultures from **(D)**. Data are shown as relative values compared to the DMSO control, which was set to 100%, ±standard derivation. **(F)** Relative mean growth rate of cultures from **(D)**. Data are indicated as relative values compared to the DMSO control, which was set to 100%, ±standard derivation. **(G)** Representative western blot analysis of total protein extract of *P. anserina* WT (*n* = 4) and *PaMth1_OEx* cultures (OEx; *n* = 4) treated with DMSO (Con) or quercetin (Quer). **(H)** Quantification of PaMTH1 protein level normalized to the Coomassie-stained gel. Protein level in WT cultures treated with DMSO was set to 1. Error bars correspond to the standard deviation and *P*-values were determined by two-tailed Student’s *t*-test. ^∗^*P* < 0.05; ^∗∗^*P* < 0.01, ^∗∗∗^*P* < 0.001.

The quercetin-induced increase of PaMTH1 abundance suggests a role of PaMTH1 in quercetin-induced longevity. To address this possibility experimentally, we investigated the effect of quercetin in a *PaMth1* deletion mutant (*ΔPaMth1*) and a *PaMth1* overexpression strain (*PaMth1_OEx*). First, we determined the lifespan of both mutants. Strikingly, in comparison to the WT (**Figure 1B**), the lifespan of *ΔPaMth1* is not affected by quercetin treatment (**Figure 5A**). Most interestingly, the mean lifespan increasing effect of quercetin in the WT (**Figure 1C**) is not observed in the deletion mutant (**Figure 5B**, **Table 1**, and Supplementary Figure S2C). Also, this mutant exhibited no difference in relative growth rate upon quercetin treatment (**Figure 5C** and Supplementary Figure S2D). In contrast, in *PaMth1* overexpressing strains lifespan is increased by quercetin (**Figure 5D**). Interestingly, quercetin has a significantly stronger effect on mean lifespan of *PaMth1_OEx* than in the WT. In the overexpression mutant, the relative mean lifespan is increased by 13.4% (23 d vs. 20 d) (**Figure 5E**, **Table 1**, and Supplementary Figure S2E), while an increase of only 10.2% is observed in the WT (**Figure 1C**, **Table 1**). As in the WT, the growth rate of the *PaMth1* overexpression mutant is slightly reduced (**Figure 5F** and Supplementary Figure S2F). Compared to the WT treated with quercetin we found a stronger increase in the amount of PaMTH1 in the overexpression mutant (**Figures 5G,H**). Therefore, we assume that in the *PaMth1* overexpression mutant quercetin methylation is higher than the WT suggesting that the level of quercetin methylation regulated lifespan extension.

**Table 1:** Overview about the relative change in mean lifespan by quercetin and isorhamnetin in different *P. anserina* strains.

	Quercetin	Isorhamnetin
Wild type	+10.2% (21 d vs. 19 d)	+17.5% (23 d vs. 20 d)
Δ*PaMth1*	+4.9% (20 d vs. 19 d)	+13.5% (24 d vs. 21 d)
*PaMth1_OEx*	+13.4% (23 d vs. 20 d)	

Overall, our data suggest that PaMTH1 is required for quercetin-induced lifespan extension and provides new mechanistic support of earlier findings about a role of PaMTH1 in lifespan control (Kunstmann and Osiewacz, 2008, 2009; Chatterjee et al., 2015).

### PaMTH1 Affects Mitochondrial Respiration and ROS Abundance

**FIGURE 6:**
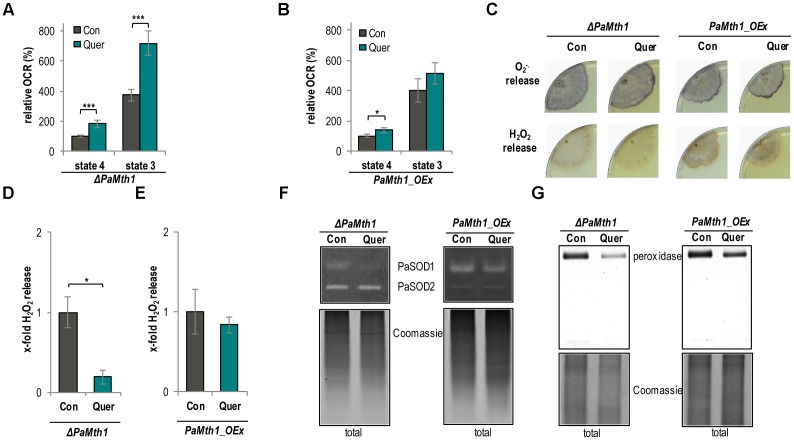
Mitochondrial function and ROS metabolism are affected depending on the methylation state of quercetin. **(A)** Complex I-dependent OCR of mitochondria from *ΔPaMth1* cultures treated with quercetin (Quer, *n* = 3 biological replicates, each with six technical replicates) compared to mitochondria from cultures treated with DMSO (Con; *n* = 3 biological replicates, each with five technical replicates). State 4 OCR of DMSO-treated mitochondria was set to 100%. **(B)** Complex I-dependent OCR of mitochondria from *PaMth1_OEx* cultures treated with quercetin (Quer, *n* = 3 biological replicates, each with five to six technical replicates) compared to mitochondria from cultures treated with DMSO (Con; *n* = 3 biological replicates, each with five technical replicates). State 4 OCR of DMSO-treated mitochondria was set to 100%. **(C)** Qualitative determination of superoxide anion release by a histochemical NBT staining and hydrogen peroxide release by a histochemical DAB staining in *ΔPaMth1* and *PaMth1_OEx* cultures (*n* = 4) treated with quercetin (Quer) or DMSO (Con). **(D)** Quantification of H_2_O_2_ release measurement from *P. anserina ΔPaMth1* cultures treated with quercetin (Quer; *n* = 4) compared to DMSO (Con; *n* = 4) treatment. The mean release of the DMSO-treated cultures was set to 1. **(E)** Quantification of H_2_O_2_ release measurement from *P. anserina PaMth1_OEx* cultures treated with quercetin (Quer; *n* = 4) compared to DMSO (Con; *n* = 4) treatment. The mean release of the DMSO-treated cultures was set to 1. **(F)** Representative “in-gel” SOD (PaSOD1 and PaSOD2) activity staining and **(G)** “in-gel” peroxidase activity staining from total protein extract of *ΔPaMth1* and *PaMth1_OEx* cultures treated with quercetin (Quer; *n* = 4) or DMSO (Con; *n* = 4). Error bars correspond to the standard deviation and *P*-values were determined by two-tailed Student’s *t*-test. ^∗^*P* < 0.05, ^∗∗^*P* < 0.01, ^∗∗∗^*P* < 0.001.

To experimentally analyze the impact of quercetin and PaMTH1 on aging and lifespan control in some more detail, we next investigated the effect of the methylation of quercetin on respiration in *PaMth1* mutants. Unexpectedly, we found that, in comparison to the WT, quercetin treatment leads to a higher increase of the OCR in both analyzed respiration states in the *PaMth1* deletion mutant (**Figure 6A** and **Table 2**). In concordance, the OCR of the overexpression mutant is less affected compared to the WT (**Figure 6B** and **Table 2**).

**Table 2:** Overview about the relative changes in the respiration states of *P. anserina* WT and mutants with the different supplementations compared to the respective DMSO control.

	Quercetin (%)		Isorhamnetin (%)	
	State 4	State 3	State 4	State 3
Wild type	+47	+41	+30	+7
Δ*PaMth1*	+83	+93		
*PaMth1_OEx*	+40	+29		

Next, we analyzed whether or not ROS levels are influenced by the methylation of quercetin. We histochemically examined the superoxide anion and hydrogen peroxid release of both *PaMth1* mutants. Compared to the WT we found a higher release of superoxide anions in the deletion mutant. In the overexpression mutant the release is hardly affected (**Figure 6C**). In contrast, hydrogen peroxide is lowered in the *PaMth1* deletion strain and rather unaffected in the overexpression strain (**Figure 6C**). We quantified hydrogen peroxid release and observed a significant lower release in the deletion mutant and no change in the overexpression strain (**Figures 6D,E**) suggesting a methylation-dependent effect of quercetin on the activity of the superoxide scavenging enzyme PaSOD1. This conclusion is supported by a strongly decreased PaSOD1 activity in *ΔPaMth1*, and a slightly reduced activity in *PaMth1_OEx* after quercetin treatment (**Figure 6F**). Like in the WT, these activity changes do not result from differences in the amount of PaSOD1 but rather from post-translational activation (Supplementary Figure S4). The lowered hydrogen peroxide release seems to affect the activity of the hydrogen peroxide scavenging peroxidase. Quercetin treatment leads to a strongly reduced peroxidase activity in *ΔPaMth1* and a slight decrease in *PaMth1_OEx* (**Figure 6G**).

### Isorhamnetin Increases the Lifespan of *P. anserina*

**FIGURE 7:**
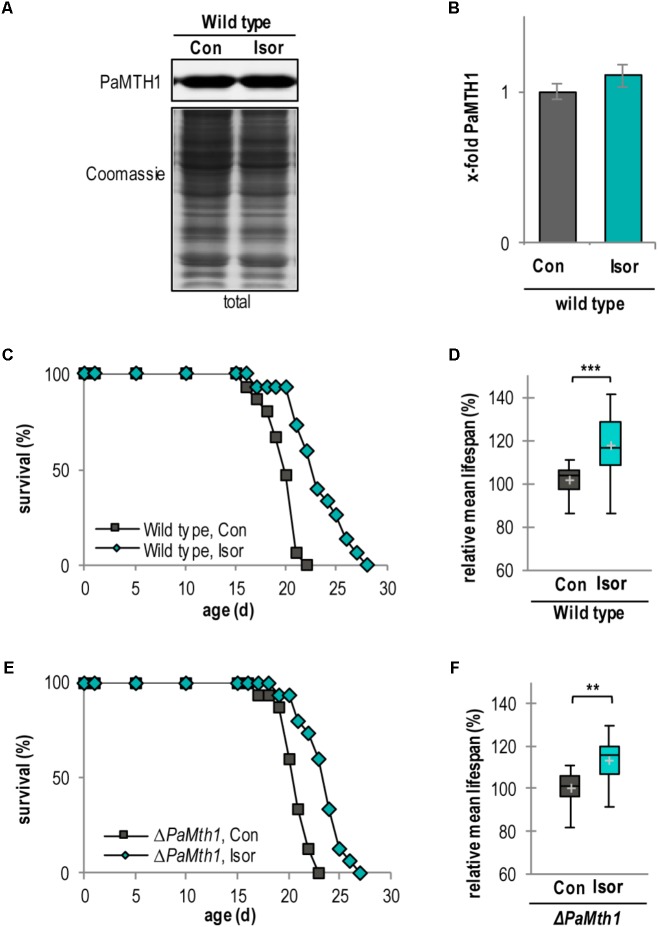
Isorhamnetin increases lifespan of the WT and *PaMth1* deletion mutant. **(A)** Representative western blot analysis of total protein extract of *P. anserina* WT cultures treated with DMSO (Con; *n* = 4) and isorhamnetin (Isor; *n* = 4), respectively. **(B)** Quantification of PaMTH1 protein level normalized to the Coomassie-stained gel. Protein level in the DMSO control was set to 1. Protein level in the DMSO control was set to 1. **(C)** Lifespan of *P. anserina* WT cultures grown on standard M2 medium containing DMSO (Con; total *n* = 15) or isorhamnetin (Isor; total *n* = 15; *P* = 0.00016). **(D)** Lifespan of *ΔPaMth1* cultures grown on standard M2 medium containing DMSO (Con; total *n* = 15) or isorhamnetin (Isor; total *n* = 15; *P* = 0.00045). **(E)** Relative mean lifespan of cultures from **(A)**. Data shown as relative values compared to the DMSO control, which was set to 100%. **(F)** Relative mean lifespan of cultures from **(B)**. Data shown as relative values compared to the DMSO control, which was set to 100%. Error bars correspond to the standard deviation and *P*-values of the survival curves were determined by SPSS with three different tests and for mean lifespan by two-tailed Student’s *t*-test. ^∗∗^*P* < 0.01, ^∗∗∗^*P* < 0.001.

To confirm the impact of methylation of quercetin on aging and lifespan control we next investigated the impact of isorhamnetin, a methylated metabolite of quercetin, on the lifespan of *P. anserina*. Compared to quercetin the amount of PaMTH1 is not affected by isorhamnetin (**Figures 7A,B**). Similar to quercetin (**Figure 1B**), isorhamnetin increases the lifespan of the WT (**Figure 7C**). Compared to the DMSO control, the mean lifespan is significantly increased by 17.5% (23 d vs. 20 d; **Figure 7D** and Supplementary Figure S5A). Strikingly, this mean lifespan increase is significantly more pronounced by treatment with isorhamnetin (+17.5%) than with quercetin (+10.2%; **Table 1**) suggesting that lifespan extension is dependent on the methylation state of quercetin. This is confirmed by data obtained from the *PaMth1* deletion mutant. Unlike quercetin, for which methylation depends on endogenous PaMTH1, isorhamnetin is independent of this enzyme. Consistently, lifespan increase is observed in the absence of the enzyme (**Figure 7E**). Compared to the DMSO control, the mean lifespan is increased by 13.5% (24 d vs. 21 d) (**Figure 7F**, **Table 1**, and Supplementary Figure S5B).

**FIGURE 8:**
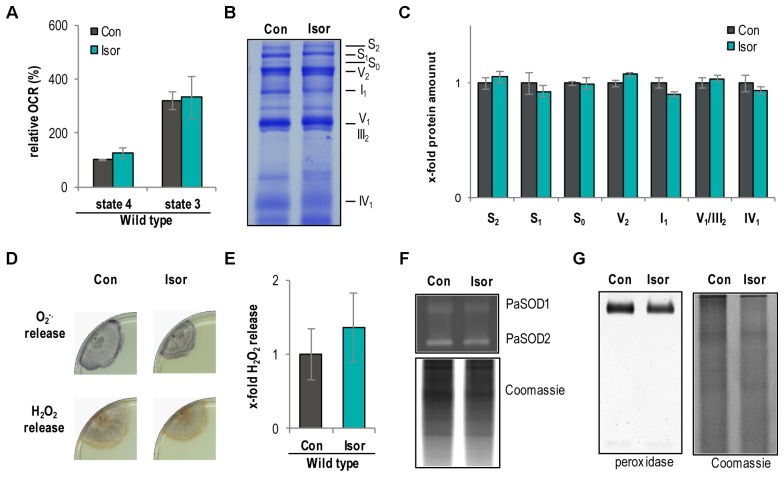
Isorhamnetin do not affect respiration and ROS metabolism of the *P. anserina* WT. **(A)** Complex I-dependent OCR of mitochondria from WT cultures treated with isorhamnetin (Isor, *n* = 4 biological replicates, each with three to six technical replicates) compared to mitochondria from DMSO-treated cultures (Con; *n* = 3 biological replicates, each with five technical replicates). State 4 OCR of DMSO-treated mitochondria was set to 100%. **(B)** Representative BN-PAGE analysis of isolated mitochondria from WT cultures treated with isorhamnetin (Isor) compared to DMSO (Con) treated. The CI_1_CIII_2_CIV_0-2_ (S_0-2_) supercomplexes, dimeric complexes III and V (III_2_ and V_2_) as well as monomeric complexes I_1_, IV_1_, and V_1_ are visualized by Coomassie staining. **(C)** Densitometric quantification of relative protein levels of ETC complexes and supercomplexes in mitochondrial extracts from isorhamnetin-treated cultures compared to DMSO-treated cultures. Optical densities of the different complexes were normalized to the total Coomassie staining of the corresponding lane. The mean abundance in the DMSO control was set to 1. **(D)** Qualitative determination of superoxide anion and hydrogen peroxide release in *P. anserina* WT cultures (*n* = 4) treated with isorhamnetin (Isor) or DMSO (Con) by a histochemical staining protocol using NBT and DAB. **(E)** Quantification of H_2_O_2_ release measurement from *P. anserina PaMth1_OEx* cultures treated with quercetin (Quer; *n* = 4) compared to DMSO (Con; *n* = 4) treatment. The mean release of the DMSO-treated cultures was set to 1. **(F)** Representative “in-gel” SOD (PaSOD1 and PaSOD2) activity staining, **(G)** representative “in-gel” peroxidase activity staining from total protein extract of *P. anserina* WT cultures treated with isorhamnetin (Isor) or DMSO (Con).

Next, we analyzed the impact of isorhamnetin on respiration, the respiratory chain, and ROS abundance. Treatment with isorhamnetin did not show any effect on complex I-dependent OCR of WT mitochondria (**Figure 8A**). This unchanged respiration suggested that the composition of the respiratory chain is unchanged as well. Indeed, isorhamnetin did not change the composition of the respiratory chain as seen in the BN-PAGE (**Figure 8B**). Quantification of protein bands revealed that the amount of respiratory chain complexes and mtRSCs was unchanged (**Figure 8C**). Also, the analysis of the ROS metabolism provides no differences in strains treated with isorhamnetin or DMSO. In detail, the release of superoxide anions and hydrogen peroxide (**Figures 8D,E**) as well as the activity of PaSOD1 (**Figure 8F**) and of peroxidase is not affected (**Figure 8G**). These data support the findings from investigations with the *PaMth1* mutants that methylated quercetin affects lifespan and not the OCR and the ROS abundance.

## Discussion

In our effort to unravel mechanistic insights about the role of natural compounds used to intervene into aging processes, we found that supplementation of the growth medium with 300 μM quercetin leads to an increase in mean and maximal lifespan of *P. anserina*. These data are in line with previously published findings in *C. elegans*, *S. cerevisiae*, and *D. melanogaster* (Belinha et al., 2007; Kampkötter et al., 2008; Saul et al., 2008; Pietsch et al., 2009). Similar to the 10% lifespan extension in *P. anserina*, Surco-Laos et al. (2011) also found a lifespan extension by 10% in *C. elegans* after treatment with quercetin. Most strikingly, in *P. anserina* this effect is linked to an increased abundance of PaMTH1, an enzyme that previously was found to accumulate in total cell extracts and in mitochondria during aging (Averbeck et al., 2000; Groebe et al., 2007; Kunstmann and Osiewacz, 2008). An *in-vitro* analysis revealed an *S*-adenosylmethionine-dependent *O*-methyltransferase activity of PaMTH1 which methylates vicinal hydroxyl groups in polyphenols like myricetin and quercetin (Kunstmann and Osiewacz, 2008; Chatterjee et al., 2015). Overexpression of *PaMth1* leads to an increased lifespan while deletion of the gene resulted in a decreased resistance against oxidative stress and a shortened lifespan (Kunstmann and Osiewacz, 2008, 2009).

In the current study we show for the first time that the *S*-adenosylmethionine-dependent *O*-methyltransferase PaMTH1 is required for quercetin-induced lifespan extension. Particularly, data from investigations of *PaMth1* mutants revealed a methylation-dependent quercetin effect on lifespan. Following supplementation of the growth medium with the methylated quercetin derivative isorhamnetin supports the conclusion that effects of quercetin treatment are depending on the methylation of this flavonoid. While quercetin does not affect lifespan of the *PaMth1* deletion mutant, isorhamnetin leads to lifespan extension. In the WT, isorhamnetin has a significantly greater effect than quercetin. However, although not statistically different, there is a marginal difference found in the *PaMth1* deletion mutant. We cannot completely exclude that this difference results from the activity of other *O*-methyltransferases or that unmethylated quercetin also has some minor effect on lifespan. Regardless, the PaMTH1 *O*-methyltransferase, which in a previous *in vitro* assay was shown to effectively methylate quercetin (Kunstmann and Osiewacz, 2008; Knab and Osiewacz, 2010), is responsible for the observed lifespan extension of our study. Furthermore, the association of quercetin, PaMTH1, and methylation is supported by data from *C. elegans* showing that transcript-levels of two different *O*-methyltransferases are upregulated upon treatment with quercetin or the polyphenol tannic acid (Pietsch et al., 2012). Also, previous studies with *C. elegans* showed that methylated derivatives of quercetin or epicatechin have a more pronounced effect on lifespan than the unmethylated versions (Surco-Laos et al., 2011, 2012). However, the *C. elegans* study did not experimentally link the effect of an *O*-methyltransferase-dependent methylation, the dietary application of quercetin and lifespan.

Apart from the methylation-dependent effects of quercetin, our study shows that not all quercetin effects are caused by methylated derivatives and linked to the lifespan-extending effect of quercetin. In particular, we found that the effect on OCR and the pro-oxidative effects, the decreased PaSOD1 activity and the increased superoxide release, are based on the unmethylated quercetin without a link to lifespan extension. In concordance with the role of *O*-methylation in the prevention of pro-oxidative effects (Cao et al., 1997; Lemanska et al., 2004; Duenas et al., 2010), we found that isorhamnetin has no pro-oxidative capacity like quercetin. From these data we conclude that unmethylated quercetin leads to a decline of components of the cellular ROS scavenging system (e.g., PaSOD1) as seen in the WT and the *PaMth1* deletion mutant.

Despite the increase of superoxide anion release, we obtained an enhanced OCR resulting from an increase in mtRSCs upon quercetin treatment of the WT. Normally, an increase in mtRSCs is known to improve electron flow (Schägger and Pfeiffer, 2000; Bianchi et al., 2003, 2004) and thereby reduces the generation of superoxide anion (Lenaz et al., 2010; Ghelli et al., 2013; Maranzana et al., 2013). Hence, the increase of OCR and mtRSCs (S_1_ and S_2_) abundance is a possible kind of compensation mechanism to prevent a superoxide anion generation in addition to the generation through unmethylated hydroxyl residues of quercetin.

Our observation that methylation-dependent effects of quercetin may also apply to higher eukaryotes including humans are supported by different studies in pigs, rodents, and humans. Previous investigations revealed that after oral application of quercetin mostly methylated quercetin metabolites accumulated in blood plasma and in different organs (Zhu et al., 1994; Manach et al., 1998, 2005; Ader et al., 2000; Boyle et al., 2000; Cermak et al., 2003; Hubbard et al., 2003). For instance in humans, after oral application of quercetin, 21% of this flavonoid circulating in the plasma was present as the methylated metabolite isorhamnetin (Day et al., 2001). In rats, this percentage (86%) was even higher (Morand et al., 1998; Manach et al., 1999). In line with our findings and with findings from other organisms, Niklas et al. (2012) exhibited an increased longevity in human cell cultures by quercetin treatment. In contrast to our study, this lifespan increasing effect was not linked to methylated derivative of quercetin. This role of a methyltransferase is now an important issue with perspectives for the use of quercetin in therapies to intervene into human aging. For instance, one approach could be to induce a more efficient cellular methylation capacity of quercetin via the induction of genes coding for specific *O*-methyltransferases. Another possible strategy is a direct diet with isorhamnetin, a strategy that currently is a very costly treatment. Investigations with model systems like *P. anserina* may help to unravel more mechanistic details about effective triggers in this field of research and to develop realistic interventions into aging processes.

## Author Contributions

HO conceived and supervised this study. VW and SH performed the experiments. VW, SH, and HO analyzed the data. HO and VW wrote the manuscript. All authors read the final version of the manuscript.

## Conflict of Interest Statement

The authors declare that the research was conducted in the absence of any commercial or financial relationships that could be construed as a potential conflict of interest.
