# Supplementary material for: Quercetin-Induced Lifespan Extension in Podospora anserina Requires Methylation of the Flavonoid by the O-Methyltransferase PaMTH1
Source: Front Genet. 2018 May 4;9:160. doi: 10.3389/fgene.2018.00160 (PMC5945814; doi:10.3389/fgene.2018.00160)
Supplement: Supplementary file 1 [file Data_Sheet_1.pdf]

Supplementary Material

Quercetin-induced lifespan extension in *Podospora anserina* requires methylation of the flavonoid by the *O*-methyltransferase PaMTH1

Verena Warnsmann, Saskia Hainbuch, Heinz D. Osiewacz*

*** Correspondence:** Prof. Dr. Heinz D. Osiewacz, Osiewacz@bio.uni-frankfurt.de

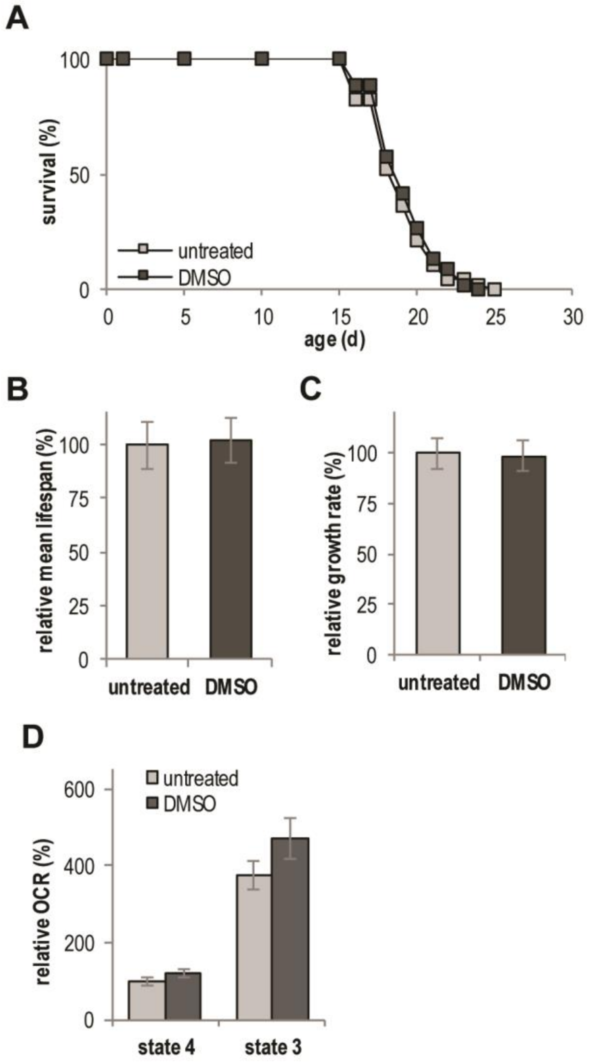

Figure S1: Lifespan and respiration of *P. anserina* wild type are not affected by the solvent DMSO. (A) Lifespan of *P. anserina* wild-type cultures grown on standard M2 medium containing DMSO (Con; n=45) compared to untreated cultures (n = 46). **(B)** Relative mean lifespan and **(C)** relative mean growth rates of *P. anserina* wild-type cultures treated with DMSO compared to untreated cultures. **(D)** Complex I-dependent oxygen consumption rate (OCR) of mitochondria from wild-type

cultures treated with DMSO (n = 3 biological replicates, each with 3-4 technical replicates) compared to mitochondria from untreated wild-type cultures (n = 3 biological replicates, each with 4-6 technical replicates). State 4 OCR of DMSO treated mitochondria was set to 100 %. Error bars correspond to the standard deviation

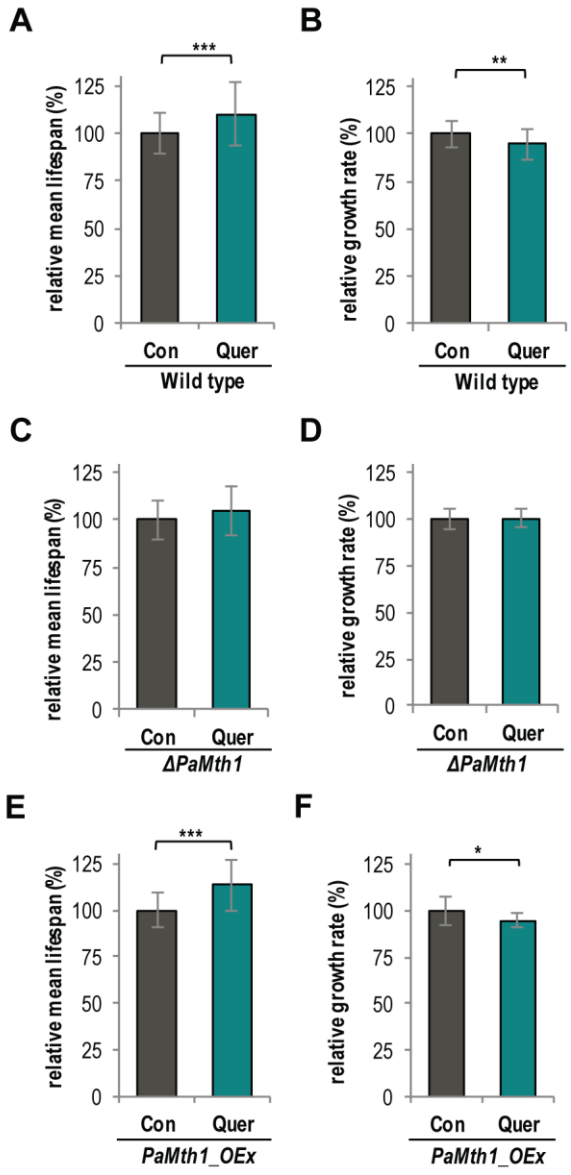

Figure S2: Mean Lifespans and growth rates of *P. anserina* wild type and *PaMth1* mutants. (A) Relative mean lifespan and **(B)** relative growth rate of *P. anserina* wild-type cultures grown on standard M2 medium the solvent DMSO as control (Con; n = 45) or quercetin (Quer; n = 46). **(C)** Relative mean lifespan and **(D)** relative growth rate of Δ*PaMth1* cultures grown on standard M2 medium the solvent DMSO as control (Con; n = 34) or quercetin (Quer; n = 31). **(E)** Relative mean lifespan and **(F)** relative growth rate of *PaMth1_OEx* cultures grown on standard M2 medium the solvent DMSO as control

(Con; n = 25) or quercetin (Quer; n = 25). Error bars correspond to the standard deviation and *P*-values were determined by two-tailed Student's *t* test. $*P < 0.05$; $**P < 0.01$; $***P < 0.001$.

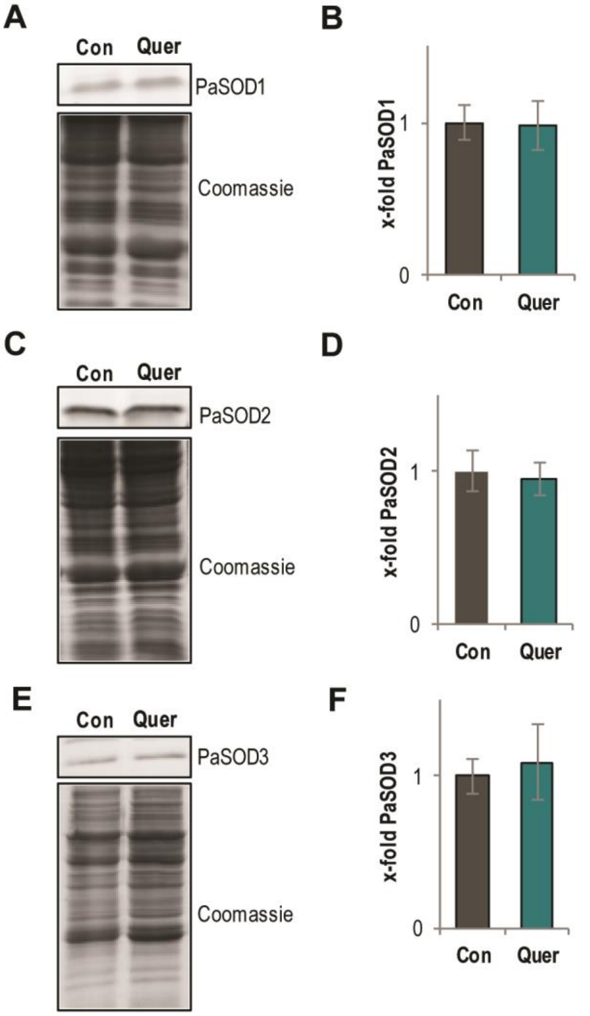

Figure S3: SOD protein levels are not affected by quercetin in *P. anserina* wild type. (A+C) Representative western blot analysis of total protein extract from *P. anserina* wild-type cultures treated with quercetin or DMSO. **(B+D)** Quantification of PaSOD1 and PaSOD2 protein level normalized to the Coomassie stained gel. Protein level in DMSO treated cultures was set to 1, respectively. **(E)** Representative western blot analysis of mitochondrial protein extract from *P. anserina* wild-type cultures treated with quercetin or DMSO **(F)** Quantification of PaSOD3 protein level normalized to the Coomassie stained gel. Protein level in DMSO treated cultures was set to 1, respectively. Error bars correspond to the standard deviation

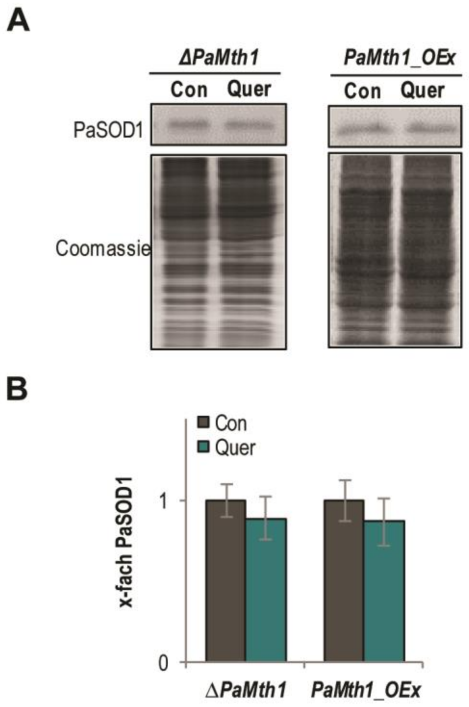

Figure S4: SOD1 protein levels are not affected by quercetin in the methyltransferase mutants. (A) Representative western blot analysis of total protein extract from *P. anserina PaMth1* mutants treated with quercetin or DMSO. **(B)** Quantification of PaSOD1 protein level normalized to the Coomassie stained gel. Protein level in DMSO treated cultures was set to 1, respectively. Error bars correspond to the standard deviation.

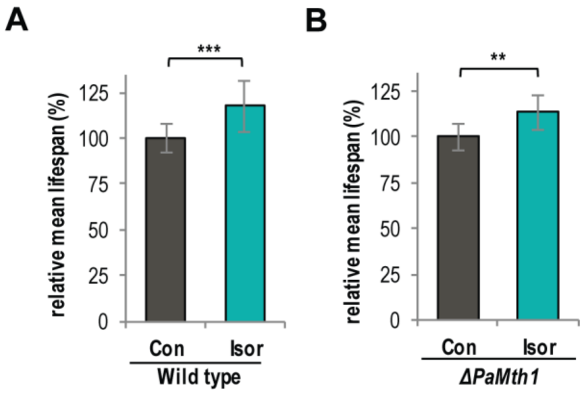

Figure S5: Mean Lifespan of *P. anserina* wild type and *PaMth1* deletion mutant treated with isorhamnetin. (A) Relative mean lifespan of *P. anserina* wild-type cultures grown on standard M2 medium the solvent DMSO as control (Con; n = 15) or isorhamnetin (Isor; n = 16). **(B)** Relative mean lifespan of Δ*PaMth1* cultures grown on standard M2 medium the solvent DMSO as control (Con; n = 15) or isorhamnetin (Isor; n = 15). Error bars correspond to the standard deviation and *P*-values were determined by two-tailed Student's *t* test. ***P* < 0.01; ****P* < 0.001.

Table S1: P-values of survival curve analysis with SPSS

	Breslow	Long rank	Tarane-Ware
**WT Quer vs. WT Con**	0.00085	0.00024	0.00049
**WT Isor vs. WT Con**	0.00016	0.00005	0.00008
**WT Isor vs. WT Quer**	0.02362	0.07608	0.03670
**Δ*PaMth1* Quer vs. Δ*PaMth1* Con**	0.13878	0.10954	0.10647
**Δ*PaMth1* Isor vs. Δ*PaMth1* Con**	0.00045	0.00008	0.00019
***PaMth1_OEx* Quer vs. *PaMth1_OEx* Con**	0.00012	0.00005	0.00007
***PaMth1_OEx* Quer vs. WT Quer**	0.01090	0.06380	0.02194
